# Preliminary Observations on the Efficacy of Efgartigimod Therapy in Autoimmune Nodopathy

**DOI:** 10.1111/ene.70269

**Published:** 2025-07-10

**Authors:** Hongfei Tai, Songtao Niu, Hua Pan, Fan Jian, Zhenxian Hu, Na Chen, Ying Wang, Zaiqiang Zhang

**Affiliations:** ^1^ Department of Neurology Beijing Tiantan Hospital, Capital Medical University Beijing China; ^2^ China National Clinical Research Center for Neurological Diseases Beijing China

**Keywords:** autoimmune nodopathy, efgartigimod, FcRn antagonist, IgG subclass, treatment response

## Abstract

**Background:**

Autoimmune nodopathy exhibits suboptimal responses to conventional immunotherapies. This study investigates the efficacy and safety of efgartigimod, a neonatal Fc receptor blocker, in this condition.

**Methods:**

A prospective single‐center study enrolled four antibody‐confirmed autoimmune nodopathy patients receiving weekly efgartigimod (10 mg/kg) over 4 weeks. Disease progression was assessed using validated neurological scales (INCAT, ISS, I‐RODS, and MRC) at baseline (Week 0), weekly during treatment (Weeks 1–4), and 4‐week post‐treatment follow‐up (Week 8).

**Results:**

Four patients (3 females, aged 17–72) responded to efgartigimod within 2 weeks, showing varied improvement based on antibody subtype. Patient 1 (anti‐NF186 IgG3+) achieved full remission by Week 2 (INCAT 3 → 0). Patient 2 (anti‐NF155 IgG4+) improved progressively (MRC 112 → 119; I‐RODS 36 → 40). Patient 3 (anti‐NF155 IgG1/IgG4+) quickly stabilized gait in the first week and gradually recovered (INCAT 5 → 2). Patient 4 (anti‐CNTN1 IgG1/IgG2/IgG3/IgG4+) reduced tremors rapidly and improved sensorimotor function (ISS 8 → 6; I‐RODS 12 → 14) despite a treatment interruption due to a fracture. Antigen‐specific efficacy varied: NF186 neuropathy resolved completely, while IgG4‐dominant paranodal cases (NF155/CNTN1) partially recovered, prompting sequential B‐cell‐targeted strategies. No severe adverse events occurred.

**Conclusions:**

Efgartigimod provided rapid functional recovery in autoimmune nodopathy. Differential responses by IgG subclass and antigenic targets highlight the necessity for biomarker‐guided strategies.

## Graphical abstract

Efgartigimod induced rapid functional improvement in four patients with autoimmune nodopathy, achieving complete remission in anti‐NF186 IgG3+ neuropathy and partial recovery in IgG4‐dominant paranodal subtypes (anti‐NF155/CNTN1+). The kinetics of response were associated with the specific antibody targets and the IgG subclass involved. The treatment was well‐tolerated, suggesting that FcRn blockade constitutes a promising therapy for this conventional treatment‐resistant disorder. Image created with BioRender.com, with permission.

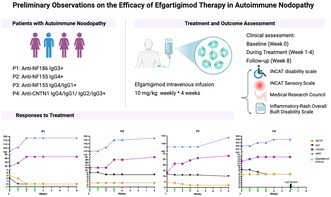

## 1 Introduction

Autoimmune nodopathy is now recognized as a distinct disorder within autoimmune peripheral neuropathies, characterized by antibodies targeting nodal/paranodal adhesion molecules crucial for axoglial integrity, such as neurofascin‐140/186 (NF140/186) at the node of Ranvier, and neurofascin‐155 (NF155), contactin‐1 (CNTN1), and contactin‐associated protein 1 (Caspr1) at paranodes [1]. The predominant antibody subclass is immunoglobulin G (IgG) 4, followed by IgG3, IgG1, and IgG2 [2]. Unlike chronic inflammatory demyelinating polyneuropathy (CIDP), autoimmune nodopathy does not show demyelination or inflammatory infiltrates. Instead, IgG4‐mediated paranodal antibodies cause myelin loop detachment and nodal elongation, while nodal antibodies lead to Schwann cell microvilli loss and decreased nodal length [3]. These distinct features prompted its reclassification as a separate disorder in the 2021 European Academy of Neurology/Peripheral Nerve Society (EAN/PNS) CIDP guidelines [4].

Clinically, autoimmune nodopathy exhibits specific antibody‐related phenotypes. Anti‐NF140/NF186 antibodies (IgG3/IgG4) correlate with acute/subacute severe sensorimotor polyneuropathy, while anti‐NF155 (predominantly IgG4) associates with chronic distal weakness, ataxia, and tremor in younger patients. Anti‐CNTN1 and anti‐Caspr1 antibodies are often found in older individuals with aggressive neuropathy and cranial nerve involvement [1]. The IgG subclass affects treatment responses, with IgG4 not activating complement and being resistant to intravenous immunoglobulin (IVIg) [2]. Over half of nodopathy patients respond poorly to IVIg, corticosteroids, or plasma exchange [1], highlighting the necessity for targeted therapies.

Recent advances in IgG4‐driven pathogenesis have led to biologics targeting autoantibody production, including B‐cell depletion, proteasome inhibition, and Fc receptor (FcRn) blockade [5]. FcRn, which regulates IgG homeostasis, can be blocked to accelerate IgG degradation, showing promise in treating myasthenia gravis [6] and other IgG‐mediated conditions [7, 8]. The FcRn antagonist efgartigimod has proven effective in a phase 3 CIDP trial [9], suggesting its potential for IgG4‐predominant nodopathy. This study examines the efficacy and safety of efgartigimod for autoimmune nodopathy, highlighting its therapeutic potential for this antibody‐driven disorder.

## 2 Methods

### 2.1 Subjects

This prospective cohort study at Beijing Tiantan Hospital enrolled patients with autoimmune nodopathy from January 2024 to January 2025, confirmed by two independent neurologists using EAN/PNS CIDP guidelines [4] and Chinese consensus [10]. Exclusions were other polyneuropathy causes, recent IVIg/plasma exchange or B‐cell depletion treatments within 8 weeks, and active malignancy or uncontrolled infection. This study was approved by the Medical Ethics Committee of Beijing Tiantan Hospital, and informed consent was obtained.

### 2.2 Data Collection and Clinical Assessments

Baseline data on demographics, disease duration, symptoms, and neurophysiological profiles were collected. Participants underwent cerebrospinal fluid (CSF) analysis for cell count, protein levels, and IgG index. Nodal/paranodal antibodies and IgG subclasses were detected using a validated cell‐based assay in serum and CSF, including anti‐NF155, NF186, CNTN1, and CASPR1 antibodies.

Clinical evaluations at enrollment and follow‐ups used the Inflammatory Neuropathy Cause and Treatment (INCAT) disability scale, the Inflammatory Neuropathy Cause and Treatment sensory scale (ISS), and the Inflammatory‐Rash Overall Built Disability Scale (I‐RODS). Muscle strength was assessed using the Medical Research Council (MRC) sum score (0–120), and tremor severity with the Fahn‐Tolosa‐Marin Tremor Rating Scale (FTM‐TRS).

### 2.3 Treatment Procedures and Outcome Assessment

Patients received weekly intravenous infusion of efgartigimod (10 mg/kg) for 4 weeks, with clinical assessments at baseline (Week 0), weekly during treatment (Weeks 1–4), and at Week 8 follow‐up. Clinical improvement was defined as a 1‐point increase in INCAT, 2–4 points in MRC sum score, 4 centile points in I‐RODS, or 2 points in m‐ISS. Safety was monitored. After Week 8, clinicians could start long‐term immunosuppressants based on the patient's condition.

## 3 Results

**FIGURE 1:**
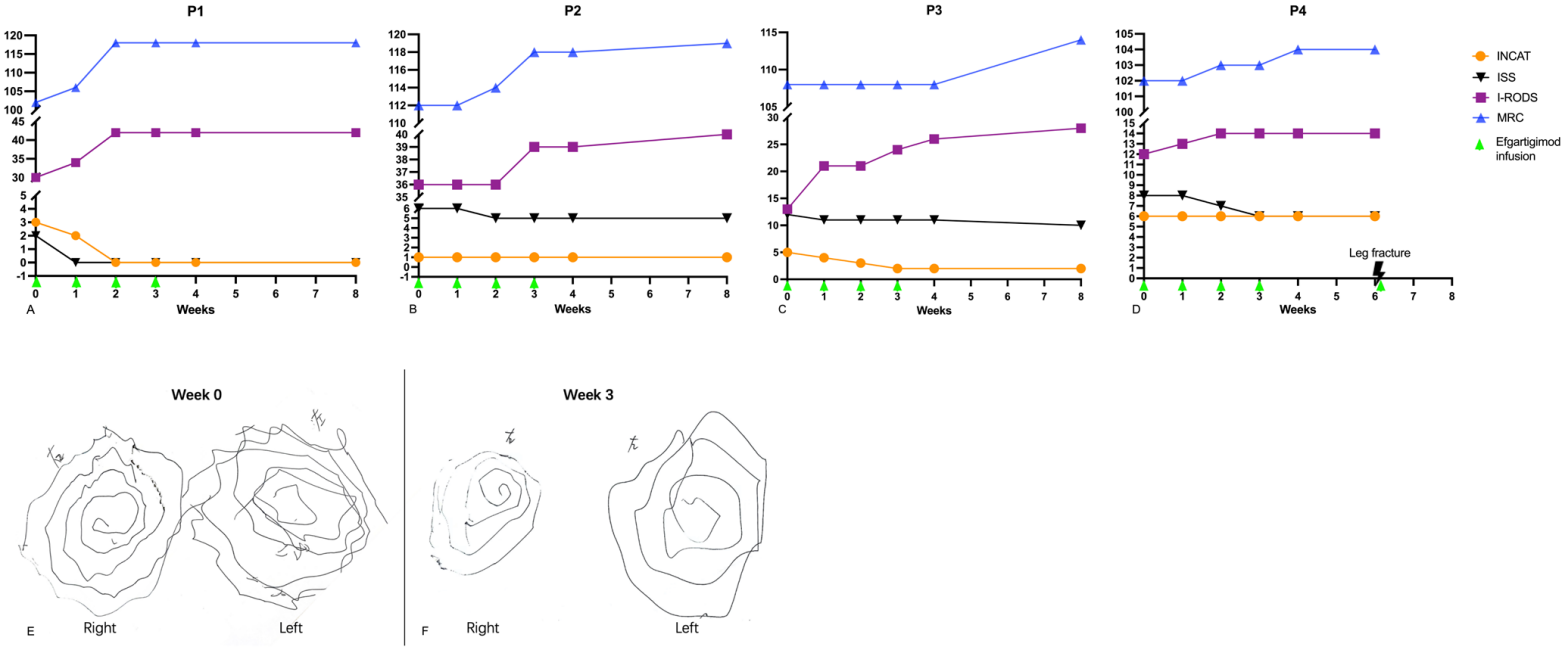
Longitudinal Assessment of Neuropathy Severity During Efgartigimod Treatment. (A–D): Serial measurements of the INCAT (Inflammatory Neuropathy Cause and Treatment) disability score, ISS (INCAT Sensory Score), I‐RODS (Inflammatory Rasch‐Built Overall Disability Scale), and MRC (Medical Research Council) sum score were conducted in four patients with autoimmune nodopathy (P1‐P4) at baseline (Week 0), during treatment (Weeks 1–4), and post‐treatment follow‐up (Weeks 8 for P1‐P3; Week 6 for P4). Arrows denote efgartigimod infusion times. (E, F) Archimedes spiral drawing test for patient 4 (anti‐CNTN1+) showed severe tremor at baseline (Week 0) with a Fahn‐Tolosa‐Marin score of 3, and reduced tremor amplitude post‐third infusion (Week 3) with a score of 2. Tests were performed under standardized conditions.

Four patients (three females, one male; aged 17–72) with autoimmune nodopathy were enrolled, targeting antibodies anti‐NF155 (*n* = 2), anti‐NF186 (*n* = 1), and anti‐CNTN1 (*n* = 1), with disease durations from 5 to 48 months. All had chronic/subacute sensorimotor polyneuropathy (Table 1). Efgartigimod led to varying clinical improvements across antibody subtypes (Figure 1).

**TABLE 1:** Clinical data of patients with autoimmune nodopathy treated with efgartigimod.

	Patient 1	Patient 2	Patient 3	Patient 4
Age (year)	35	17	51	72
Sex	Female	Male	Female	Female
Disease duration (m)	6	12	5	48 (4 m since this recurrence)
Clinical symptoms	Subacute quadriparesis and glove‐stocking hypoesthesia	Chronic muscle weakness and hypoesthesia in distal lower limbs	Chronic quadriparesis and hypoesthesia in distal limbs, sensory ataxia	Recurrent quadriparesis and hypoesthesia in distal limbs, sensory ataxia, actional tremor
Electrophysiological examination	Sensorimotor polyneuropathy with CB	Sensorimotor polyneuropathy with CB and TD	Sensorimotor polyneuropathy with CB	Sensorimotor polyneuropathy with CB
CSF white blood cell (number/mL)	8	4	4	2
CSF protein (g/L)	0.61	2.00	6.08	1.54
Anti‐nodal/paranodal antibodies (serum/CSF IgG subclass & titer)	Anti‐NF186 Serum: IgG3 1:100 CSF: negative	Anti‐NF155 Serum: IgG4 1:320 CSF: IgG4 1:3.2	Anti‐NF155 Serum: IgG1 1:320, and IgG4 1:100 CSF: IgG4 1:100	Anti‐CNTN1 Serum: IgG4 1:320, IgG1 1:320, IgG2 1:100, and IgG3 1:32 CSF: IgG4 1:32, IgG1 1:32, and IgG2 1:1
Comorbidity	None	None	Chronic hepatitis B (entecavir‐managed)	None
Previous immunotherapy	IVIG at 2 months prior enrollment	None	Refractory to intravenous methylprednisolone (1 g/day×5) → oral prednisone (60 mg/day×8 weeks)	IVIG and steroid during the initial onset stage; none upone recurrence
Long‐term immunosuppressive therapy after efgartigimod induction	Mycophenolate mofetil	Rituximab	Rituximab	—

Abbreviations: CB, conduction block; CNTN1, contactin‐1; CSF, cerebrospinal fluid; IgG, immunoglobulin G; IVIG, intravenous immunoglobulin; NF155, neurofascin‐155; NF186, neurofascin‐186; TD, temporal dispersion.

### 3.1 Case‐Specific Outcomes

#### 3.1.1 Patient 1 (Anti‐NF186 IgG3+)

A 35‐year‐old pregnant woman developed subacute quadriparesis and glove‐stocking hypoesthesia after a respiratory infection. She exhibited proximal upper limb weakness, impaired hand dexterity, areflexia, and sensory deficits. Cervical MRI showed nerve root hypertrophy, and serum anti‐NF186 IgG3 was detected. Lumbar puncture was not performed due to pregnancy. IVIg treatment partially improved her condition, allowing for term delivery.

Six weeks postpartum, her symptoms worsened. Initial scores were MRC 102, INCAT 3, ISS 2, and I‐RODS 30. CSF analysis showed albumin‐cytological dissociation without anti‐NF186 antibody. Efgartigimod treatment led to rapid improvement, with complete resolution after two doses (Figure 1A). A slight rebound occurred after Week 8, leading to the addition of mycophenolate mofetil and three more monthly efgartigimod infusions, achieving sustained remission.

#### 3.1.2 Patient 2 (Anti‐NF155 IgG4+)

A 17‐year‐old male experienced progressive distal sensorimotor neuropathy over 12 months, marked by leg dragging, trouble standing on tiptoe, or climbing stairs. Examination revealed muscle atrophy, weakness (MRC grade 4) in distal lower limbs, high arches, absent reflexes, and reduced sensations in the feet, with coordination issues. Whole‐exome genetic testing ruled out hereditary neuropathies. CSF analysis indicated albumin‐cytological dissociation. Both serum and CSF tested positive for anti‐NF155 IgG4 antibodies.

Efgartigimod treatment showed initial improvement by Week 2, with further recovery by Week 8, as indicated by MRC (112 → 119), ISS (6 → 5), and I‐RODS scores (36 → 40, Figure 1B), though INCAT scores remained unchanged. Long‐term low‐dose rituximab (500 mg every 6 months) treatment was initiated.

#### 3.1.3 Patient 3 (Anti‐NF155 IgG1/IgG4+)

A 51‐year‐old woman with chronic hepatitis B on entecavir developed progressive sensorimotor neuropathy over 5 months, experiencing numbness, tingling, and weakness in her hands and feet, along with severe sensory ataxia. Neurophysiological studies revealed nerve conduction abnormalities with conduction block and CSF albumin‐cytological dissociation. Despite high‐dose corticosteroid (intravenous methylprednisolone 1 g/day×5 followed by oral prednisone 60 mg/day×8 weeks), she was resistant.

Antibody tests at our center revealed anti‐NF155 antibody positive (serum: IgG1/IgG4+; CSF: IgG4+). Adding efgartigimod to prednisone (40 mg/day) rapidly improved her condition, enhancing gait stability in the first week, and gradually reducing paresthesia and weakness, as evidenced by various assessments (INCAT:5 → 2; ISS:12 → 10; I‐RODS:13 → 28; MRC:108 → 114, Figure 1C). After Week 8, low‐dose rituximab therapy was initiated.

#### 3.1.4 Patient 4 (Anti‐CNTN1 IgG1/IgG2/IgG3/IgG4+)

A 72‐year‐old woman experienced acute‐onset quadriplegic neuropathy with glove‐stocking numbness 4 years ago, worsening to bedridden status and respiratory issues within 2 weeks. Initial tests showed CSF albumin‐cytological dissociation and sensorimotor polyneuropathies with conduction block. IVIg provided temporary relief, but symptoms returned and were resistant to further IVIg. Methylprednisolone led to gradual recovery over 18 months, leaving slight fingertip numbness.

Four months before enrollment, she experienced recurring numbness to wrists and knees, sensory ataxia, and tremor, requiring assistance to walk despite slight muscle weakness. Anti‐CNTN1 antibody was positive (serum: IgG4/IgG1/IgG2/IgG3+; CSF: IgG4/IgG1+).

Efgartigimod therapy improved her tremor (FTM‐TRS: 32.5 → 25.5), numbness, muscle strength, and functional scores (ISS: 8 → 6, MRC: 102 → 104; I‐RODS: 12 → 14, Figure 1D–F). In Week 7, she fell, fractured her tibia and fibula, required surgery and bed rest, and received an extra efgartigimod dose to prevent perioperative exacerbation.

## 4 Discussion

This study provides the first clinical evidence that FcRn blockade with efgartigimod is an effective treatment for autoimmune nodopathy. By examining four cases, we found: (1) a universal early response within 2 weeks, surpassing conventional therapies; (2) response kinetics linked to IgG subclasses and antibody targets, with IgG3/NF186 cases showing rapid, complete relief; while IgG4‐dominant paranodal cases showed partial improvement and needed additional B‐cell depletion for sustained remission.

Efgartigimod offers a new treatment option by disrupting IgG recycling for rapid antibody clearance without involving complement activation or cellular immunity [11]. This is particularly beneficial in IgG4‐dominant nodopathy, where conventional treatments like corticosteroids and IVIg are less effective [12]. Our findings show that even treatment resistant IgG4 autoantibodies can be affected by FcRn blockade.

There is a hierarchy in IgG subclasses responses (IgG3 > IgG1 > IgG4), aligned with their biological traits [13]. IgG3, with its high FcRn affinity and short half‐life, enables rapid clearance, as seen in patient 1's fast symptom relief. IgG4 can form bispecific antibodies and convert with IgG1‐3, potentially sustaining nerve damage and requiring prolonged treatment, as in patients 2, 3, 4.

Treatment efficacy varied between nodal (NF186) and paranodal (NF155/CNTN1) autoantibody targets. The NF186 patient recovered fully in 2 weeks, while paranodal cases showed only partial improvement. Structural biology indicates that NF186's location at nodes may facilitate antibody–antigen dissociation, whereas anti‐NF155/CNTN1 antibodies cause structural damage in paranodes, with node elongation and Kv channels dislocation, complicating repair. This spatial difference underscores the necessity for antigen‐stratified treatments. Notably, the anti‐NF186 case in this study involved IgG3‐subclass autoantibody, suggesting that both target specificity and IgG subclass influence treatment outcomes.

Efgartigimod shows rapid effectiveness within two weeks, faster than rituximab's three‐month onset [14, 15]. This is consistent with the ADHERE trial in CIDP, where efgartigimod showed benefits within two to four weeks [9], making it suitable for induction therapy for autoimmune nodopathy exacerbations. Our findings support a precision medicine approach, customizing treatments based on IgG subclass profiles and antigenic targets. Efgartigimod induction can lead to early improvement, especially in patients with IgG3/IgG1 dominance. While IgG4‐dominant cases also improved early, the response is partial and sustained. In the maintenance phase, patients with paranodal anti‐NF155/CNTN1+ and IgG4 dominance may benefit from prolonged efgartigimodand long‐term B‐cell depletion (e.g., rituximab), potentially enhancing symptom improvement and reducing disabilities, which requires further study.

This study sheds light on FcRn‐targeted therapy for autoimmune neuropathy, but the findings should be interpreted cautiously due to limitations. The small cohort (*n* = 4) restricts efficacy analysis of antigen targets and subclass interactions, and the 8‐week follow‐up is insufficient for assessing long‐term outcomes. Future studies should include larger, multicenter cohorts, extended follow‐up, and control treatments to address these issues.

In summary, the study highlights efgartigimod's potential in treating autoimmune nodopathy, emphasizing rapid symptom relief and tailored strategies based on antibody subclasses and antigen targets. The results suggest FcRn blockade is a safe, fast‐acting therapy, with further research needed to enhance long‐term outcomes through stratified aproaches.

## Author Contributions

**Hongfei Tai:** conceptualization, investigation, writing – original draft, formal analysis, methodology, data curation, software. **Songtao Niu:** conceptualization, methodology, supervision. **Hua Pan:** conceptualization, investigation, methodology. **Fan Jian:** methodology, investigation. **Zhenxian Hu:** investigation. **Na Chen:** investigation. **Ying Wang:** investigation. **Zaiqiang Zhang:** methodology, conceptualization, writing – review and editing, supervision, project administration.

## Conflicts of Interest

The authors declare no conflicts of interest.

## Data Availability

Anonymized data that support the findings of this study will be available from the corresponding author upon reasonable request.
